# Calcite moonmilk of microbial origin in the Etruscan *Tomba degli Scudi* in Tarquinia, Italy

**DOI:** 10.1038/s41598-018-34134-y

**Published:** 2018-10-26

**Authors:** Angela Cirigliano, Maria Cristina Tomassetti, Marta Di Pietro, Francesco Mura, Maria Lorella Maneschi, Maria Donatella Gentili, Barbara Cardazzo, Chiara Arrighi, Cristina Mazzoni, Rodolfo Negri, Teresa Rinaldi

**Affiliations:** 1 La Sapienza University of Rome, Dept. of Biology and Biotechnology Charles Darwin, Rome, 00185 Italy; 2 Freelance restorer, Via Flavia 16, 00062, Bracciano, Rome, Italy; 3 Research Center for Nanotechnology Applied to The Engineering of Sapienza (CNIS), La Sapienza University of Rome, Rome, 00185 Italy; 4 Archaeologist, Capo Delegazione FAI – Delegazione Viterbo, Via XX Settembre 56, 01016 Tarquinia, Italy; 5 Archaeologist, Via de Carolis 135, 00136 Rome, Italy; 6 Department of Comparative Biomedicine and Food Science, University of Padova, Viale dell’Università, 16, Legnaro, 35020 Padova, Italy; 7 Freelance restorer, Via San Maria Mediatrice 10, 00165 Rome, Italy

## Abstract

A white deposit covering the walls in the *Stanza degli Scudi* of the *Tomba degli Scudi*, Tarquinia, Italy, has been investigated. In this chamber, which is still preserved from any kind of intervention such as cleaning and sanitization, ancient Etruscans painted shields to celebrate the military power of the Velcha family. Scanning electron microscopy analysis has revealed the presence of characteristic nanostructures corresponding to a calcite secondary mineral deposit called moonmilk. Analysis of the microbial community identified *Proteobacteria*, *Acidobacteria* and *Actinobacteria* as the most common phyla in strong association with the moonmilk needle fibre calcite and nanofibers of calcium carbonate. Employing classical microbiological analysis, we isolated from moonmilk a *Streptomyces* strain able to deposit gypsum and calcium carbonate on plates, supporting the hypothesis of an essential contribution of microorganisms to the formation of moonmilk.

## Introduction

**Figure 1:**
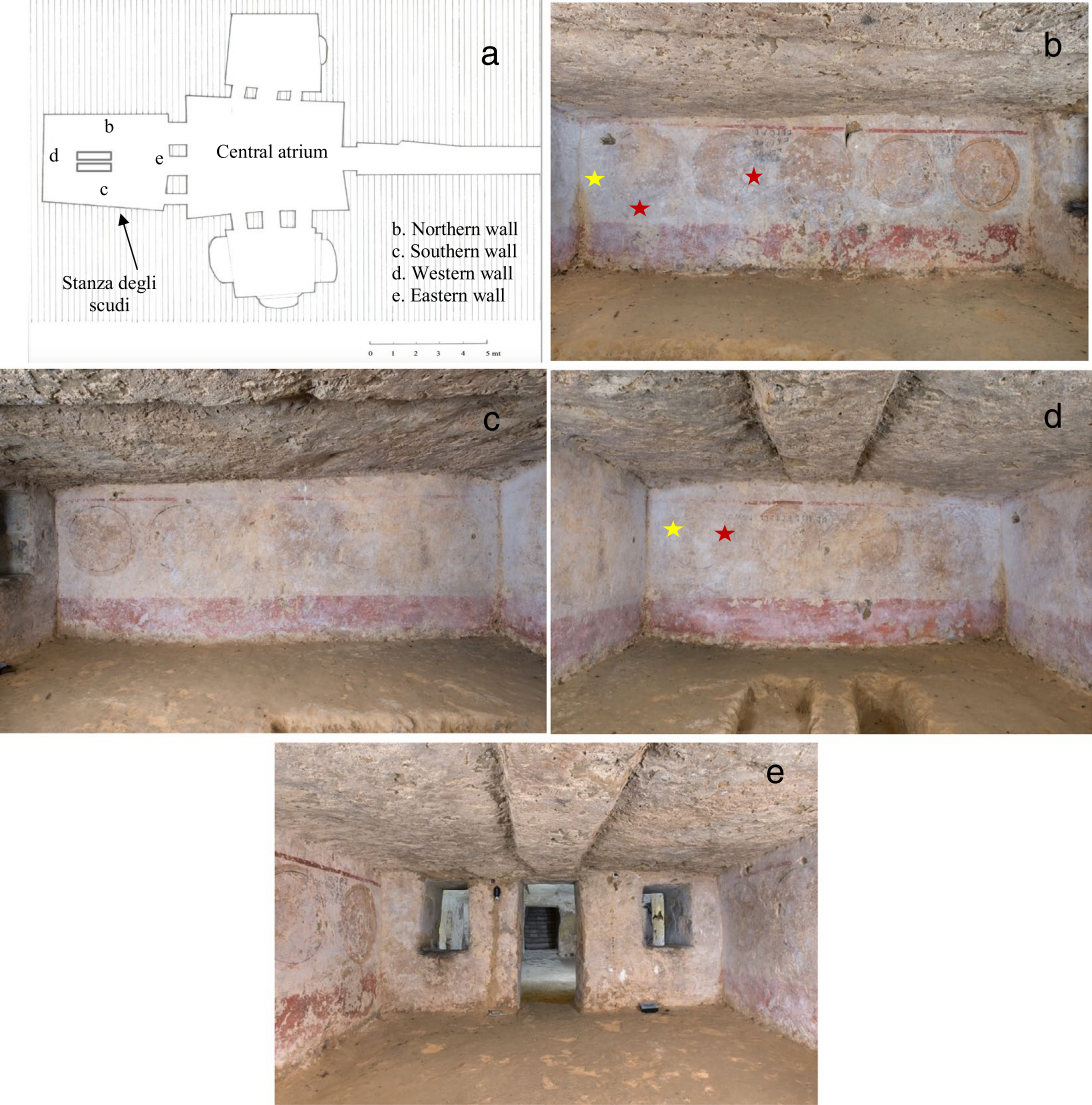
Etruscan *Tomba degli Scudi*, Tarquinia, Italy. (**a**) Hand-drawn map of the tomb from Maria Cristina Tomassetti, based on measurements taken during the restoration. The walls of the *Stanza degli Scudi* are indicated with letters. (**b**) Picture of the northern wall, (**c**) southern wall, (**d**) western wall, (**e**) and eastern wall. The red stars mark the sampling sites for microbiological analysis, and the yellow stars indicate the metagenomics sampling sites. A fine and uniform dry white *patina* is present on the wall surfaces with a gradient from the western wall towards the central atrium. The eastern wall has less *patina* than the other walls. Photographer: Domenico Ventura.

The *Tomba degli Scudi*, dated to the late classical period (mid-IVth century B.C.)^1^, has been designated a UNESCO site since 2004 and is located in the Monterozzi necropolis of Tarquinia. This tomb is considered one of the largest tombs in Tarquinia, and its structure simulates an Etruscan house with a central atrium into which three chambers open^2,3^ (Fig. 1a). The name of the tomb signifies the frieze in the burial chamber, which depicts fourteen shields painted in yellow ochre with black contours and aims to highlight the predominant role of the Velcha clan in the military field (Fig. 1b). In the central atrium, important inscriptions are painted, primarily referring to the great power of the Tarquinian *gens* Velcha^4^. Moreover, in the same atrium and in the burial chamber, other inscriptions are traced which bear the names of clan members, suggesting family ties and the exact place where the individuals were buried. In the central atrium, the most prominent members of the family, including the founder of the tomb, are represented. These members include Larth Velcha, his wife, Velia Seithiti, and his parents, Velthur Velcha and Ravnthu Aprthnai^3^. The restoration of the frescoes in the central atrium, promoted by *Soprintendenza Archeologia, Belle Arti e Paesaggio*, was recently completed. In the central atrium, the walls are covered with a white deposit, corresponding to calcium carbonate (CaCO_3_, calcite), and we previously analysed the role of microorganisms in this deposit. The presence of a calcium carbonate deposit on the frescoes was one of the issues faced by the restorers. White layers of different consistency were present on the surface of the frescoes; there was a soft deposit (present on the frescoes only in the left corner of the western wall) and a thick and hard layer, which was more difficult to remove. The cleaning of the frescoes was performed using chemical (ionic exchange resins in cationic form, used at pH 5,5 to 6) and mechanical techniques (soft bristle brushes, fibreglass pen and scalpel)^4^.

In this work, we investigated the microbial activity in the burial chamber called *Stanza degli Scudi*, which was preserved from any intervention, such as cleaning or sanitization, during the restoration project of 2016 to 2017, and we describe for the first time the presence of a secondary calcite deposit, called moonmilk^5^. Moonmilk, which is a rather soft deposit when wet but resembles talcum or chalk powder when it is dry, has been used since ancient times for its putative medicinal value^6^. Moonmilk consists of unusually fine crystals of nanomeric-scale calcium carbonate and is found in karst caverns^7–9^ or in other hypogeal environments, such as lava tubes^10^ or granitic underground tunnels^11^.

The analysis of the moonmilk sampled in the not-yet-restored burial chamber in the *Tomba degli Scudi* revealed that the deposit is intimately connected with a complex microbial community, containing, in particular, *Actinobacteria* species, supporting the hypothesis of a determinant biological contribution to this characteristic calcite formation^7,8,12–17^.

## Results

### Investigation of the white deposit in the *Stanza degli Scudi*

The historical documentation of the Monterozzi necropolis reported that the *Tomba degli Scudi* was never restored in the past^18^. The first restoration project of the atrium chamber was performed in 2016–2017. Although the burial chamber, *Stanza degli Scudi*, was not included in this restoration project, a general assessment of the conditions and a microbiological analysis were carried out throughout the restoration period of the atrium chamber. In particular, the *Stanza degli Scudi* was monitored from March 2017 to December 2017; a constant temperature of 16 °C and humidity of 99–100% were registered (see Methods and Fig. S1). Figure 1a shows the map of the tomb, and Fig. 1b–e show pictures of the walls of the burial chamber.

**Figure 2:**
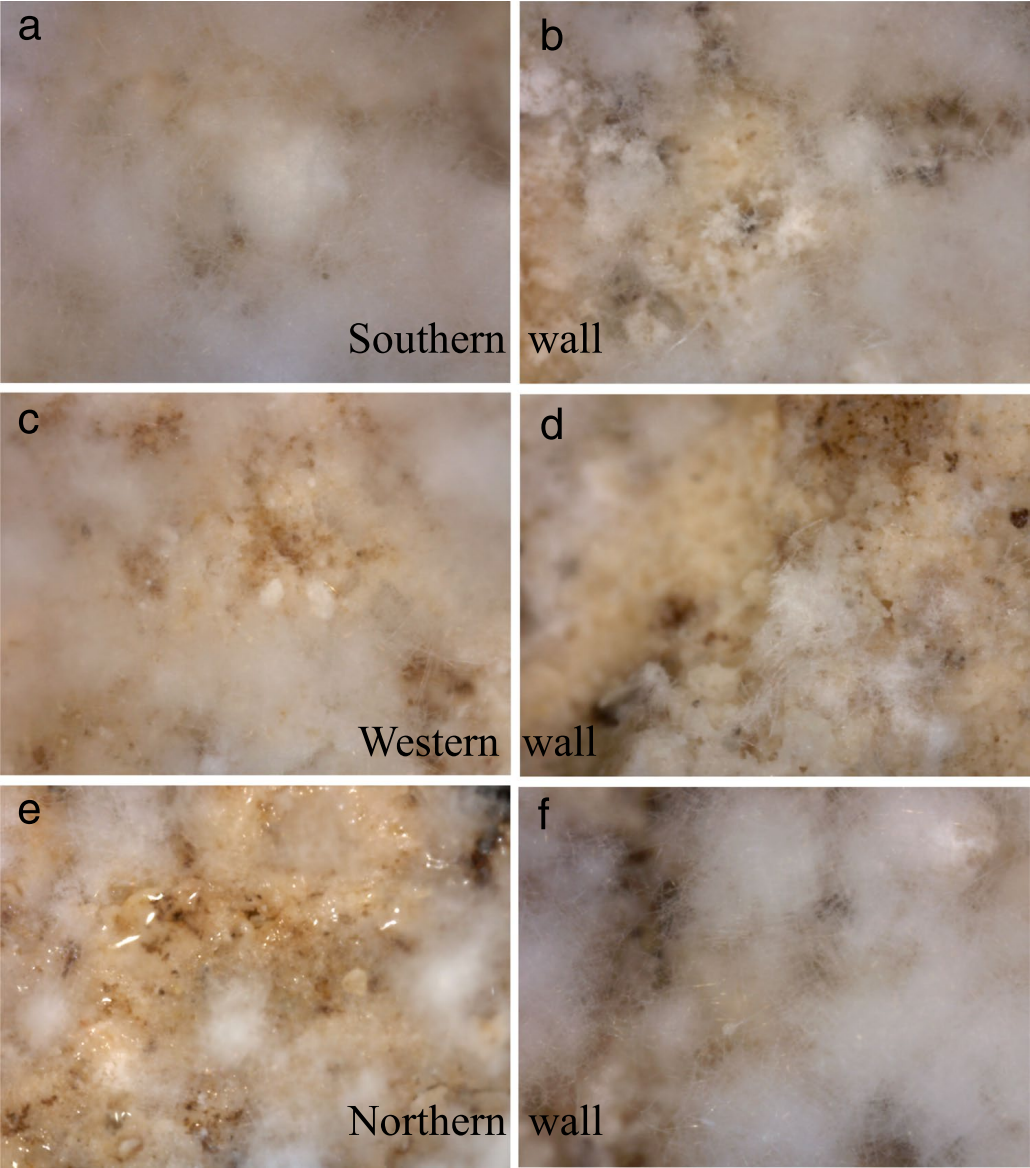
Pictures of the white deposit in the *Stanza degli Scudi*. (**a**,**b**) Southern wall. (**c**,**d**) Western wall. (**e**,**f**) Northern wall. Of note, in picture (**e**), a biofilm was identified. Images were obtained directly from the walls with a video microscope.

Only restorers, archaeologists and biologists were authorized to enter the *Tomba degli Scudi* throughout the restoration period, except during the “*giornate*” FAI (*Fondo Ambiente Italiano*), which were specific days when visitors were admitted for a short period of time, following all precautions to avoid contamination and temperature fluctuations in the tomb. Moreover, these visitors remained in the central atrium and were not admitted to the other chambers. From June-October 2016 and March-December 2017, the periods of restoration of the central atrium frescoes, we visualized the white deposit on the walls in the *Stanza degli Scudi* directly with a video microscope (Fig. 2) to assess the frescoes; the pictures revealed a white, fluffy deposit with a consistency that differed from that on the central atrium frescoes^4^. This soft deposit was noted in the atrium chamber only in the left corner of the western wall frescoes and on the ceiling. Notably, on the northern wall, Fig. 2(g), a biofilm was also visualized.

**Figure 3:**
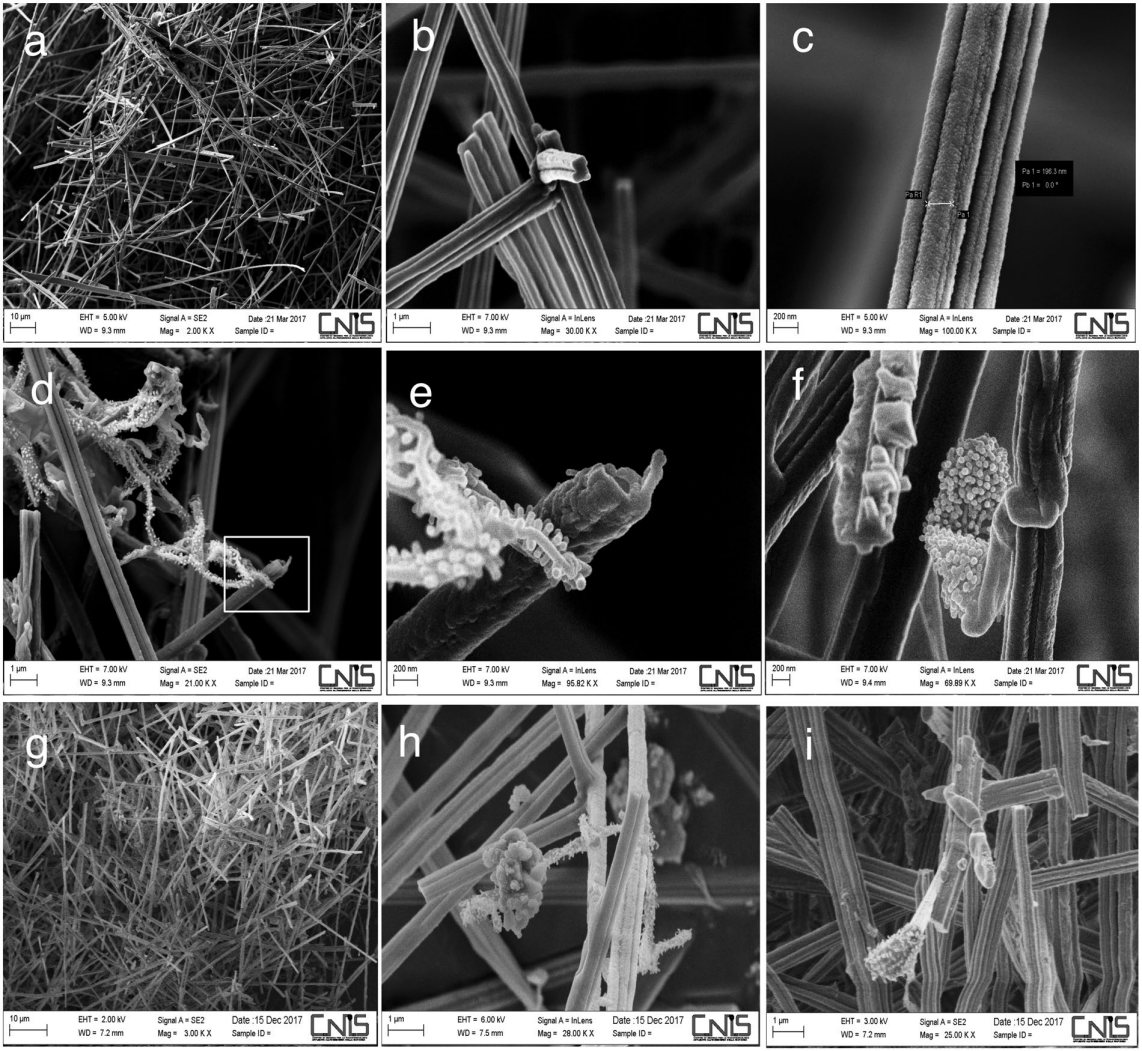
Moonmilk deposit in the Etruscan *Stanza degli Scudi*, scanning electron microscopy (SEM). (**a**–**c**) Samples taken from the western wall (indicated with the letter d in Fig. 1a) in March 2017 were composed of needle fibre calcite and calcite nanofibres (moonmilk). (**d**–**f**) In the moonmilk samples from March, a microorganism, presumably *Actinobacterium*, was present; (**e**) is a magnification of picture (**d**). Note the nanoscale dimension of the mycelium. (**g**–**i**) Samples taken from the northern wall (indicated with the letter b in Fig. 1a) in December 2017. Of note, in (**h**), a microorganism was present similar to that identified in March on the western wall (compare (**d**), (**e**) and (**h**)). In (**i**), another microorganism, presumably an *Actinobacterium* strain, was present in the moonmilk.

The white *patina* is uniformly present in the walls of the burial chamber, although it is less abundant in the eastern wall, where the entrance to this room is located. The *patina* is spread over the entire surface on the pictorial layer, and its variable thickness bears no relation to the underlying pigments. By this general assessment, we found that the patina in the *Stanza degli Scudi* was distinct from the *patina* that was removed from the atrium chamber frescoes^4^; this difference prompted the decision to investigate samples from the burial chamber with scanning electron microscopy (SEM). Figure 3 shows pictures of samples taken in March 2017 from the western wall (Fig. 3a,b and c) and in December 2017 from the northern wall (Fig. 3g): a uniform deposit was observed in both cases, and this type of calcium carbonate deposit corresponds to fibres of calcite (Fig. S2). Comparing the images obtained with those present in the literature, we concluded that this deposit is a secondary calcite deposit known as moonmilk^5^. Moonmilk is defined as being formed by needle fibres (1–2 μm) and nanofibers (less than 1μm) of calcite^16^. We identified the same morphologies of needle fibre calcite, represented in Fig. 3, that were described by Cailleau *et al*. in 2009^16^; indeed, the dimensions of the fibres are less than 1 μm, and the surfaces are smooth. One important aspect of moonmilk is that its formation has been associated with the presence of microorganisms, in particular *Actinobacteria*^19,20^. A biogenic origin of moonmilk was proposed, and *Streptomyces* species were deemed responsible for this deposit^7,13,14,17^. Indeed, we also observed microorganisms (Fig. 3(d–f),(h) and (i)) that were strongly associated with the moonmilk, which could correspond to *Actinobacteria* with a nanoscale size (see also Fig. S3).

### Isolation of bacterial species from moonmilk

We proceeded with a classical microbiological isolation technique using a fraction of the same sample observed with SEM to isolate pure cultures of microorganisms from moonmilk. The moonmilk samples from March and December 2017 were streaked directly onto YPD and LB plates (see Methods), and after 2 weeks of growth at 28 °C, we obtained strains of actinobacteria, bacilli and cocci; pure cultures of these microorganisms were observed under the microscope (examples in Fig. S4) and tested on B4-C and YPD plates supplemented with urea to verify whether the strains were able to dissolve or produce calcium carbonate, respectively. Strain 27 was able to dissolve calcium carbonate (Fig. S5a), and strain 2 was able to deposit calcium carbonate on plates (Fig. S5c and below).

**Figure 4:**
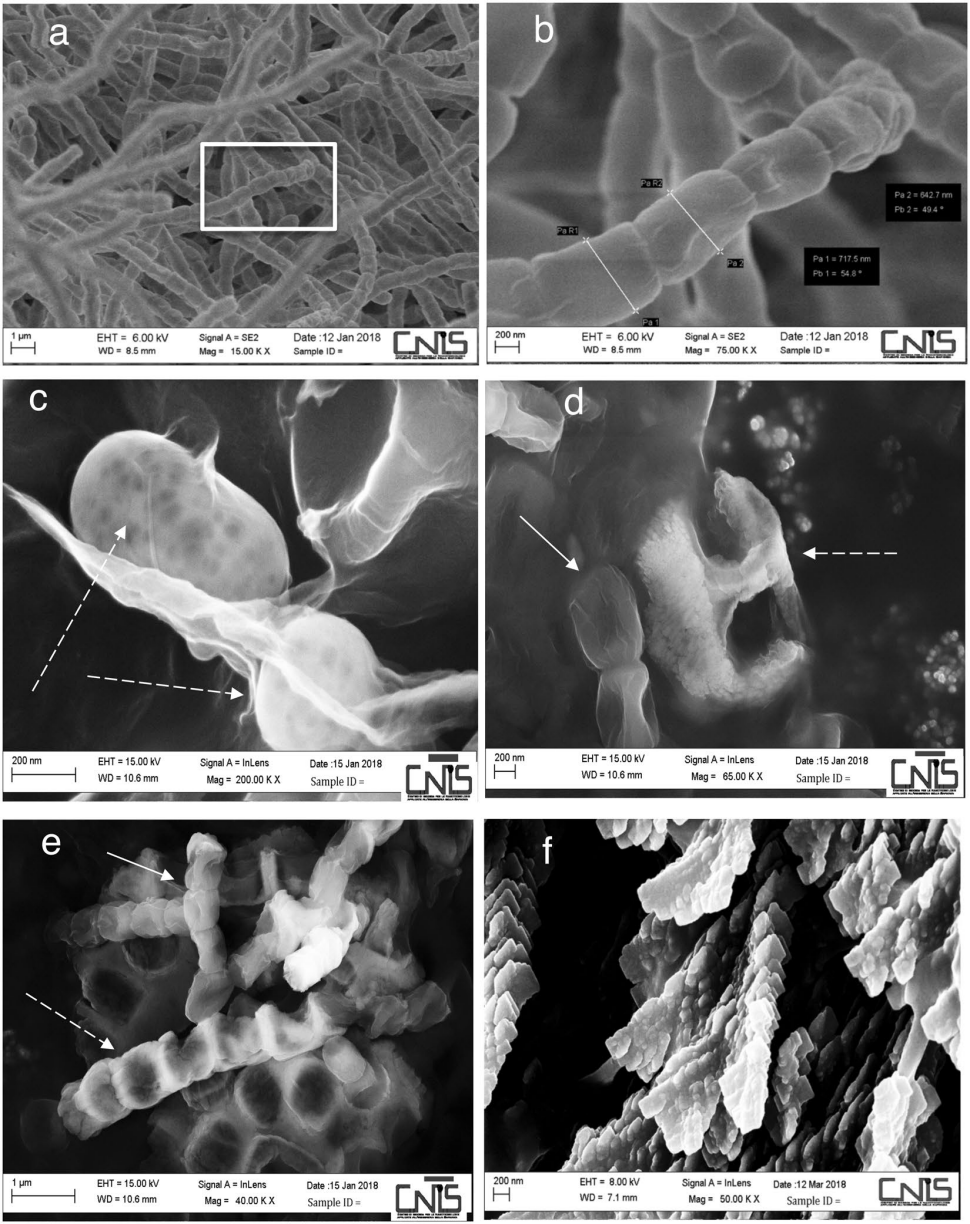
Pure culture of *Streptomyces* sp. TR2 from the *Stanza degli Scudi*, grown in different media and observed by scanning electron microscopy (SEM). (**a**) Purified mycelium of *Streptomyces* sp. TR2 isolated from western wall moonmilk and grown at 18 °C on a YPD-rich medium plate. (**b**) Magnification of the spore chain morphology of *Streptomyces* sp. TR2 in the white square in (**a**); the diameters of the two spores are 642,7 nm and 717,15 nm (**c**–**e**) A pure culture of *Streptomyces* sp. TR2 grown in synthetic medium supplemented with CaCO_3_ without glucose, is producing calcium sulfate (see Figure S4c). (**c**) Spheroidal elements (dashed white arrow) corresponding to calcium sulfate are embedded in the mycelium (see also Figure S5). (**d**) A spore chain (white arrow) and a calcium sulfate deposit originate from the mycelium; the calcium sulfate shape could correspond to a spore chain mark. In (**e**), a spore chain (white arrow) is embedded in a calcium sulfate deposit (dashed white arrows), suggesting a complete adherence to the bacteria. (**f**) A pure culture of *Streptomyces* sp. TR2, grown in YPD medium supplemented with urea is producing calcium carbonate (see also Figures S5c and S8).

We concentrated our analysis on strain 2, which was identified as a *Streptomyces* strain and named *Streptomyces* sp. TR2; Table S1 shows the closest bacterial species relatives to this strain based on 16S gene alignment performed with the BLAST program. We also tested the growth of the *Streptomyces* sp. TR2 pure culture in various media. The strain was streaked on complete YPD medium and synthetic medium supplemented with CaCO_3_ with or without glucose as a carbon source (Fig. S6) (see Methods). Of note, the *Streptomyces* sp. TR2 strain grew more rapidly than other *Actinobacteria* strains isolated from moonmilk. Moreover, this strain was able to grow in 20 days at 18 °C in a synthetic medium supplemented with CaCO_3_ without glucose; samples of *Streptomyces* sp. TR2 from such plates were observed with SEM. Figure 4a shows the *Streptomyces* mycelium, and the magnification of a spore chain is observed in Fig. 4b. In the absence of glucose, with CaCO_3_ in the synthetic medium, *Streptomyces* sp. TR2 produced spherical crystals and deposits (indicated with a dashed white arrow in Fig. 4c and d). The atomic composition of these structures suggested the presence of an inorganic compound identified as calcium sulfate, CaSO_4_ (see Fig. S7); calcium sulfate dihydrate CaSO_4_·2H_2_O is gypsum. Of note, shown in Fig. 4d, the CaSO_4_ clearly originates from the mycelial surface, and this deposit retains the shape of the spore chains (Fig. 4e, see Discussion). To analyse the capability of the isolated *Streptomyces* sp. TR2 to deposit calcium carbonate, a pure culture of *Streptomyces* sp. TR2 was streaked on YPD medium supplemented with urea; after 8 weeks at 37 °C, colonies were observed with SEM, and the analysis revealed the production of calcium carbonate crystals (Figs 4f and S8).

### Identification of bacterial species from moonmilk via a metagenomic approach

The microbial community profiles of moonmilk samples were assessed by 16S rRNA gene sequencing using Illumina technology. First, DNA was extracted from samples collected from the western (indicated as W) and northern (indicated as N) walls of the *Stanza degli Scudi* (Fig. 1) and used as template for the library construction. From the MiSeq raw sequence data, 12,732 filtered sequences and 299 final features were obtained using the DADA2 pipeline in QIIME2. The rarefaction curves for both samples plateaued, indicating that the obtained sequencing depth was good (data not shown). The Faith’s phylogenetic diversity (a qualitative measure of community richness that incorporates phylogenetic relationships between the features) indices were 14,69 and 19,27 for the W and N samples, respectively.

**Figure 5:**
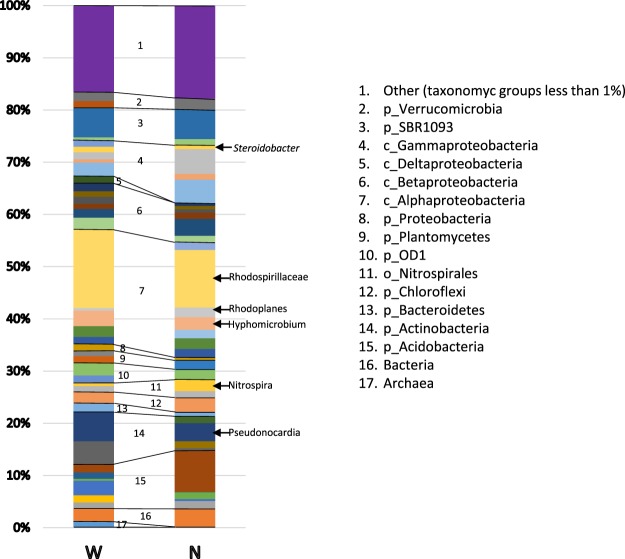
Composition of the community in two samples of moonmilk taken from the western and northern walls of the *Stanza degli Scudi*. The predominant taxa are highlighted.

The most common phylum was *Proteobacteria*, which represented approximately 50% of the population in both samples, followed by the phyla *Acidobacteria* (11.24–13.03%) and *Actinobacteria* (11.61–7.03%). The class *Alphaproteobacteria* predominated the community, forming approximately 25% of the population, followed by *Betaproteobacteria* (9.9-8.1%), *Gammaproteobacteria* (7.7–12.3%) and *Actinobacteria* (6.7-4.9%). The communities of the two samples were composed by the same phyla (considering only taxonomic groups representing more than 1% of the population), excluding the phylum *Firmicutes*, which was present only in the N sample. Most of the classes (those representing more than 1%) are represented in both samples, demonstrating that, although only one sample was analysed for each sampling position, the community may be representative of the microbiota contained in the tomb. Few sequences were identified at the genus/species level (20/299 features), defining only approximately 15% of the total population (the list is presented in Table S2). The low number of sequences classified to the genus/species level reflects a limitation of the NGS method currently available due to the reduced classification performance of short sequencing reads^21^. The most represented genus/species were *Pseudonocardia* and *Hyphomicrobium*. Figure 5 shows the composition of the two communities, highlighting the following abundant taxa: the genera *Steroidobacter*, *Hyphomicrobium*, *Rhodoplanes*, *Nitrospira* and *Pseudonocardia;* and the family *Rhodospirillaceae*.

## Discussion

When the *Tomba degli Scudi* was discovered in 1870, several tombs of the Monterozzi necropolis had already been discovered and studied. In 1871, Professor Bompiani visited the newly discovered *Tomba degli Scudi* and reported the following observations: “on the ceiling there is a sort of white mold which, under the pressure of the fingers, melts into a milky substance”^18^. This observation fits perfectly with the description of moonmilk, indicating that moonmilk was present at the time of tomb discovery and suggesting that the equilibrium of the chemical-physical conditions and the presence of microorganisms in the tomb resulted in the production of moonmilk. Nevertheless, when the atrium chamber was restored, we did not identify moonmilk, but rather a calcite deposit with spheroidal CaCO_3_, on the frescoes^4^. The only exception was the presence of moonmilk in the corner of the western wall in the atrium chamber; in this wall, a window is open to the burial chamber (Fig. S9a and b). Due to its dry texture, moonmilk can be easily removed from the wall with a soft brush, thus we hypothesize that this deposit was easily eliminated in the past to facilitate a better appreciation of the frescoes of the atrium; this hypothesis is supported by the observation that the ceiling of the atrium chamber remains covered with moonmilk, especially near the burial chamber (Fig. S9c).

We demonstrated for the first time that a hypogeal tomb, the *Tomba degli Scudi*, contains moonmilk. In Italy, moonmilk has been described in caves in the Italian Alps^22,23^, in the *Grotta Nera* in the Appennini^24–27^, and in the Su Bentu cave in Sardinia^28^. A world map depicting all caves that have been analysed with molecular biological methods was published in 2012^29^.

Indeed, the microbial community that we isolated from moonmilk in the *Stanza degli Scudi* is composed of genera already described to be associated with moonmilk from Palaeolithic painted caves^17,20,30,31^; for example, in moonmilk from the Altamira cave, the most representative phylum was *Proteobacteria*, followed by *Actinobacteria*, which is a very similar composition to that observed in the *Stanza degli Scudi*^13^. This result also supports the biogenic origin of the calcium carbonate deposit. By SEM analysis, we visualized nanofilamentous actinobacteria in moonmilk (Fig. 3d,e,f and h and Fig. S3) that we were not able to culture under standard conditions; a method for isolating rare *Actinobacteria* has been highlighted in a report only recently^31^. From a moonmilk deposit in Belgium, several nanosized *Streptomyces* (up to 150 nanometres) were cultured, indicating the presence of nanosized bacteria in moonmilk^17^. As a comparison, the *Streptomyces* sp. TR2 exhibited a spore diameter of 700 nanometres (Fig. 4).

It was also suggested that the progressive accumulation of calcite causes microorganisms to become trapped in the crystal matrix^13^ and that carbonate precipitates could lead to the entombment of bacteria in these crystals, terminating their growth^8,17^. Indeed, we observed the same phenomenon when the *Streptomyces* sp. TR2 strain produced gypsum on plates (Fig. 4d and e). This bacterium was able to grow in minimal medium without glucose and to deposit CaCO_3_ on plates. From the moonmilk, we also selected cocci that were able to dissolve CaCO_3_ on plates (Fig. S5), revealing a delicate metabolic equilibrium between bacteria that enable the production of moonmilk in the environment.

The moonmilk microbial community deserves further investigation for possible biotechnological applications of microorganisms able to deposit or dissolve calcium carbonate. Notably, *Streptomyces* strains isolated from moonmilk may also be a promising target for the discovery of novel bioactive molecules^20,32^, especially in light of the great *Actinobacteria* diversity present in various samples from moonmilk deposits within the same cave^33^.

Why is moonmilk created in the hypogeal *Tomba degli Scudi*? Comparing our results with those of other studies, it appears that the conditions for creating moonmilk require a complex equilibrium of biological and geological factors; human intervention (both restoration and visitors) also greatly influence the stability of this equilibrium. Indeed, it has been proposed that calcification is a physiological adaptation for the removal of calcium ions (Ca^2+^) that are toxic to bacterial communities living in calcium-rich environment^34^. The metabolic activities of microorganisms create conditions that are favourable for the precipitation of calcium carbonate, generating a microenvironment with a high pH^15^. Progressive calcium carbonate precipitation results in macroscopic calcitic moonmilk deposits.

All of the conditions described as being required for the biogenesis of moonmilk in caves are present in the *Tomba degli Scudi:* a) the tomb was excavated in limestone, a fossiliferous calcarenite called *macco*^35^; b) all the preparatory and pictorial layers contain calcium, and the plaster is composed of lime and grinded limestone (*macco*); and c) the preparatory layer on which the pigments are applied is composed by slaked lime, and the paintings were made using the *fresco* technique (in this technique, pigments are dispersed in water and fixed on the surface through the carbonation process: calcium hydroxide present in the plaster combines with carbon dioxide, generating calcium carbonate, thus fixing pigments in its crystalline matrix). Thus, calcium is present in very high amounts on the walls. Moreover, other important parameters, including the temperature (16 °C) and humidity (90 to 100%) are constant. Finally, bacterial species able to precipitate CaCO_3_ were isolated.

Only the following two Etruscan tombs in which both microbial communities and calcium carbonate were investigated are described in the literature: the *Tomba della Scimmia* and *Tomba del Colle* (Chiusi, Italy)^36,37^. The *Tomba della scimmia* was restored twice (in 1993 and in 2000), and biocide treatments were performed; a recent microbial community analysis revealed the predominance of the *Actinobacteria* phylum in this tomb, in particular the genus *Pseudonocardia*^36^. The *Tomba del Colle* was also cleaned and restored twice (1993–1996 and 2003–2006) with a biocide treatment^37^. In the *Tomba del Colle*, a predominance of *Alphaproteobacteria* was reported, specifically the orders *Rhizobiales* and *Rhodospirillales*^37^. We cannot compare those results with the microbial communities analysed in this study, because in the *Stanza degli Scudi*, no restoration or biocide treatments were performed, and no visitors were admitted; rather, the human intervention in the two tombs in Chiusi appears to have selected for specific bacterial species. SEM analysis revealed the presence of spheroidal elements of CaCO_3_ in the *Tomba della Scimmia* and nest-like aggregates of calcite crystals in the *Tomba del Colle*, indicating that, at least in these specific samples, moonmilk was not present^36,37^.

In the *Tomba della Scimmia* and *Tomba del Colle*, the paintings were made on a thin clay coating that was applied directly to the walls^36,37^. We could speculate that the very low amount of calcium present in clay promotes spherical structures of CaCO_3_ rather than the deposition of needle fibre calcite and nanofibres of calcium carbonate; the structure is also presumably influenced by the microbial community.

Although *Streptomyces* species were described as being responsible for the biodeterioration of hypogeal frescoes^36,38^ and the discoloration of ancient mural painting in Egyptian tombs^39–43^, these species may actually exert a protective effect on the frescoes due to their association with moonmilk; in fact, restorers reported that under the *patina* in the *Tomba degli Scudi*, the frescoes were well preserved. In 1921, the restorer O. Paternostro was one of the first to observe that under the calcium carbonate, the colours of the painting were vivid^44^. A soft calcium carbonate deposit was already described in other tombs of the Monterozzi Necropolis (for a complete description, see Adele Cecchini, 2012^18^). Our future work will involve a general assessment of the presence of moonmilk in the Monterozzi necropolis under the supervision of Soprintendenza Archeologia, Belle Arti e Paesaggio. It will be very important to determine whether the typical physiological conditions of the tombs in the Monterozzi necropolis favour moonmilk formation and to compare these results with other studies.

In the future, moreover, the atrium chamber frescoes will be monitored to assess the sequence of microorganisms that re-colonize the walls, in light of the microbial community composition in the *Stanza degli Scudi*.

In conclusion, we reported a fruitful example of the interconnection between restoration and microbiological investigation; indeed, the unexpected presence of moonmilk in the burial chamber influences the decision for the next step of restoration, as this calcium carbonate *patina* may be protective rather than damaging to the frescoes. In light of this finding, the moonmilk may be left in place while the best restoration approach is determined.

## Methods

### Sample collection

Samples were collected on YPD plates (rich medium containing 1% Bacto-peptone, 1% yeast extract, 2% glucose and 2% agar), LB (0.5% yeast Extract, 1% Bacto-tryptone, 0.5% NaCl, 1 mL 1 N NaOH, and 2% agar), and synthetic medium (0.17% yeast nitrogen base without amino acids, 0.5% ammonium sulfate, 1% CaCO_3_ with or without 2% Glucose and 2.5% agar). Moonmilk samples were taken from the walls with sterile cotton swabs, and samples intended for SEM analysis were observed within 24 hours. Samples used for microbiological analysis were streaked in sterile conditions on YPD and LB plates, and the growth was followed for 2 weeks. The production of calcium carbonate was monitored on YPD plates containing 3 g/L urea and 25 g/L CaCl_2_ and LB plates containing 4 g/L CaCl_2_. The dissolution of calcium carbonate was monitored on B4-C medium (0.4% yeast extract, 0.5% glucose, 0.25% CaCO_3_, and 1.4% agar)^45^.

### Microscopy

Bacterial pictures were taken with a Zeiss Axio Imager Z1 Fluorescence Microscope with AxioVision 4.8 Digital Image Processing System using a 63×oil lens. Scanning electron microscopy (SEM) micrographs and EDX spectra were obtained using an FESEM Zeiss Auriga equipped with a Bruker Quantax detector. To improving the conductivity of the samples, they were coated with a 50-nm layer of gold (Fig. 4a–c) and chromium (Fig. 4d) using the Quorum Q150T ES sputter machine.

### Amplification, sequencing and analysis of the 16S-rRNA gene

A single colony for each bacterial strain was picked and suspended in 20 μL of microLYSIS buffer (Labogen, Rho, Italy). Total DNA was isolated according to the manufacturer’s instructions. Then, 2 μL of each DNA extract was used for 16S ribosomal DNA (rDNA) amplification using a standard PCR protocol with the universal bacterial primers P0 (5′-GAGAGTTTGATCCTGGCT-3′) and P6 (5′-CTACGGCTACCTTGTTAC-3′)^46^. The 16S rDNA amplification was verified by agarose gel electrophoresis. The band of approximately 1500 bp was purified (Gel/PCR DNA fragments Extraction kit, Geneaid, cat. DF 100) and sequenced with both primers by a sequencing service (http://www.biofabresearch.it/index2.html). The resulting sequences were analysed using the online BLAST program at https://blast.ncbi.nlm.nih.gov/^47^. The sequences, submitted to GenBank, received the accession number MH512911.

### Environmental Parameters

An automatic *in situ* monitoring system for measuring microenvironmental parameters (e.g., temperature, relative humidity) that was installed in the *Tomba degli Scudi* provided climatic data.

### Sampling, DNA extraction and NGS sequencing of moonmilk samples

Moonmilk for the metagenomics analysis was scraped and immediately analysed from the western and northern walls of the *Stanza degli Scudi*, at the locations indicated in Fig. 1. The two samples were denoted W and N. For the DNA extraction 2 mg of moonmilk was subjected to the protocol of the DNeasyPowerSoil kit (Qiagen, Hilden, Germany), following manufacturer’s instructions. For NGS sequencing the V3-V4 regions of the 16SrRNA gene were amplified using primers 341F and 805R^48^ modified with forward and reverse overhangs, 0.25 mM dNTPs, 1× Phusion HF buffer and 1 U Phusion high-fidelity DNA polymerase (New England Biolabs, Ipswich, MA). PCR was performed in a 2720 thermal cycler (Applied Biosystems, Waltham, MA) with 25 cycles at 95 °C for 30 s, 55 °C for 30 s and 72 °C for 45 s, followed by a final extension for 7 min at 72 °C. The PCR products were purified using the SPRIselect purification kit (Beckman Coulter, Brea, CA) and, after the bead purification, the targeted band was detected on a 1.8% agarose gel. Then, the amplicons were sent to BMR Genomics (Padova, Italy) for the introduction of barcodes, and the pooling and sequencing of the library on an Illumina MiSeq with the 2 × 300 bp paired end approach. A total of 180,281 raw reads were generated. The rRNA sequence data was processed using the software QIIME2 (2017.11). Raw sequence data files were imported into a QIIME2 artifact using the ‘SampleData [PairedEndSequencesWithQuality]’ semantic type. For sequence quality control and feature table construction, the DADA2 pipeline was employed^49^. To assign taxonomy to the sequences, a Naive Bayes classifier and the q2-feature-classifier plugin were used^50^. The Naive Bayes sklearn-based taxonomy classifier pre-fitted on the Greengenes 13_8 was employed with a default confidence value of 0.7. The raw sequence data was deposited in the SRA database with accession number SRP147457.

## Electronic supplementary material

Supplementary figures and tables
