# Supplementary material for: Calcite moonmilk of microbial origin in the Etruscan Tomba degli Scudi in Tarquinia, Italy
Source: Sci Rep. 2018 Oct 26;8:15839. doi: 10.1038/s41598-018-34134-y (PMC6203712; doi:10.1038/s41598-018-34134-y)
Supplement: Supplementary file 1 — Supplementary figures and tables [file 41598_2018_34134_MOESM1_ESM.pdf]

Supplementary Informations

Calcite moonmilk of microbial origin in the etruscan *Tomba degli Scudi* in Tarquinia, Italy

Angela Cirigliano[1°], Maria Cristina Tomassetti[2°], Marta Di Pietro[1], Francesco Mura[3], Maria Lorella Maneschi[4], Maria Donatella Gentili[5], Barbara Cardazzo[6], Chiara Arrighi[7], Cristina Mazzoni[1], Rodolfo Negri[1] and Teresa Rinaldi[1]*

[1] La Sapienza University of Rome, Dept. of Biology and Biotechnology "Charles Darwin", Rome, 00185, Italy

[2] Freelance restorer, Via Flavia 16, 00062, Bracciano, Rome, Italy

[3] Research Center For Nanotechnology Applied To The Engineering of Sapienza (CNIS) La Sapienza University of Rome, Rome, 00185, Italy

[4] Archaeologist, Capo Delegazione FAI – Delegazione Viterbo, Via XX Settembre 56, 01016, Tarquinia, Italy

[5]Archaeologist, Via de Carolis 135, 00136, Rome, Italy

[6] Department of Comparative Biomedicine and Food Science, University of Padova, Viale dell'Università, 16, Legnaro, 35020, Padova, Italy

[7]Freelance restorer, Via San Maria Mediatrice 10, 00165, Rome, Italy

* teresa.rinaldi@uniroma1.it

° These authors contributed equally to the work

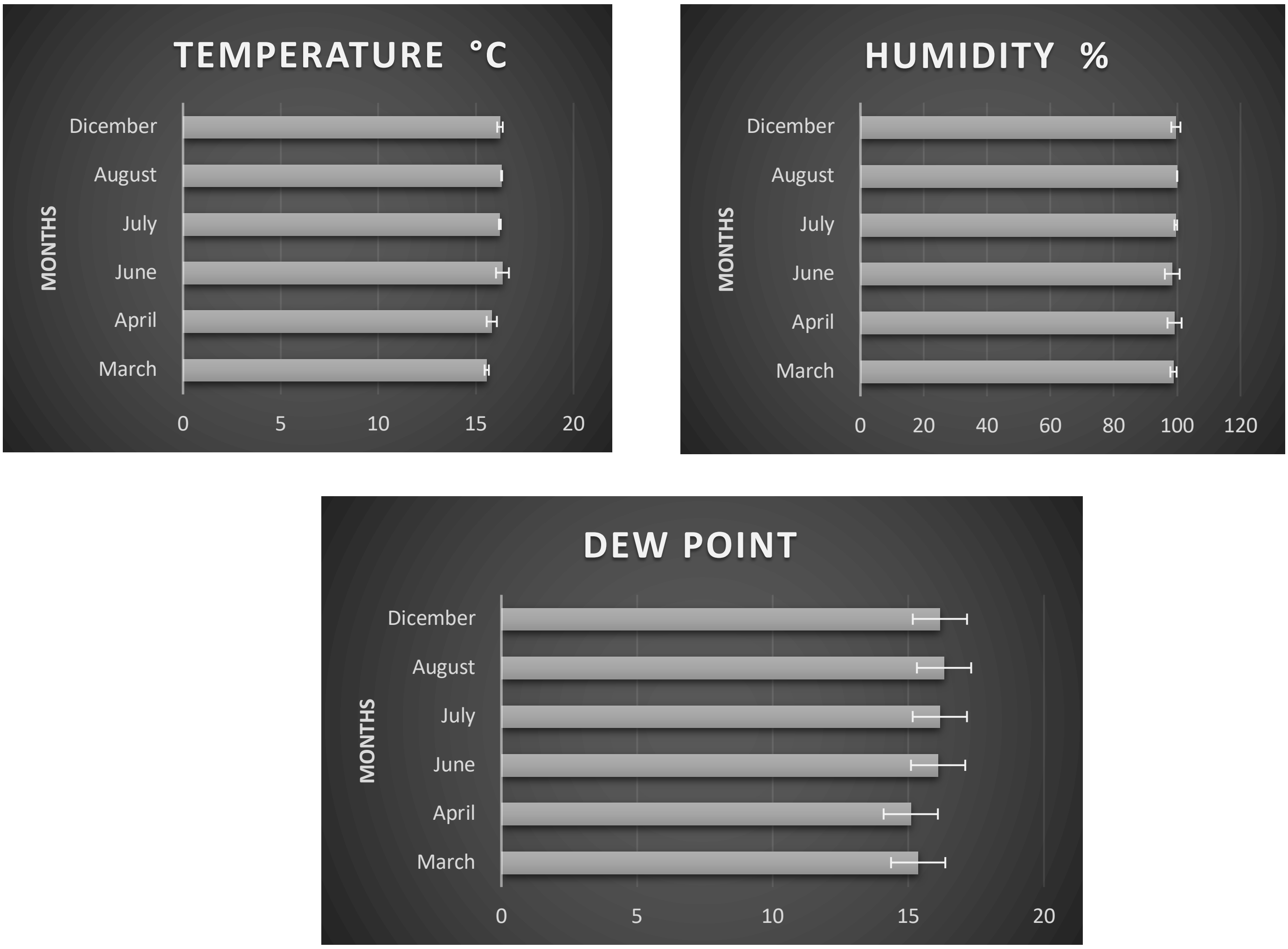

Figure S1.
Temperature, humidity and dew point registered in the *Stanza degli Scudi* in the period March 2017 to December 2017.

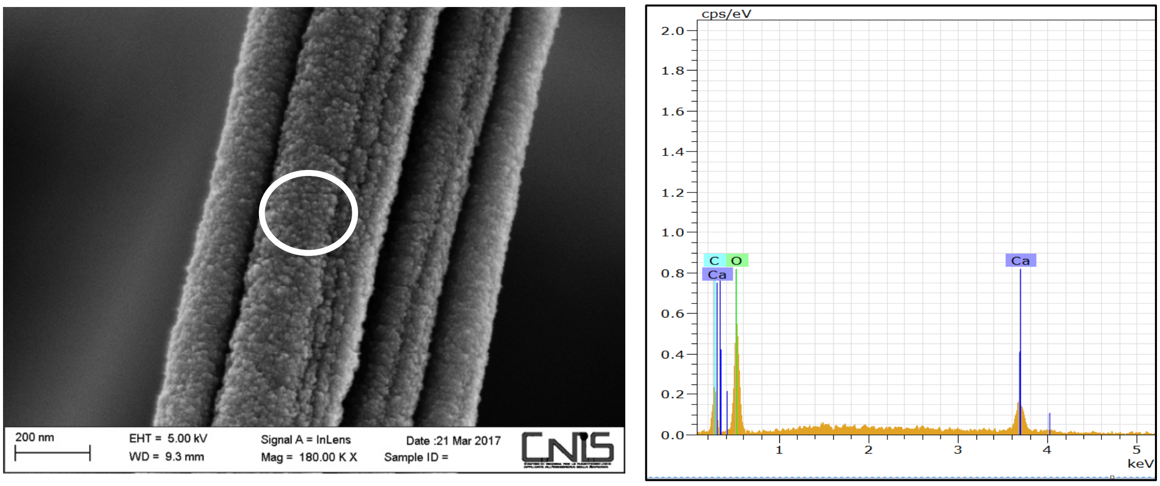

El	AN	Series	norm. C [wt.%]	Atom. C [at.%]	Error (1 Sigma) [wt.%]
O	8	K-series	43.03	58.37	1.58
Ca	20	K-series	48.45	26.24	0.58
C	6	K-series	8.52	15.39	0.44
		Total:	100.00	100.00	

Figure S2.
SEM analysis of nanofibres. The atomic composition of the deposit in Figure 3 suggests a calcium carbonate composition.

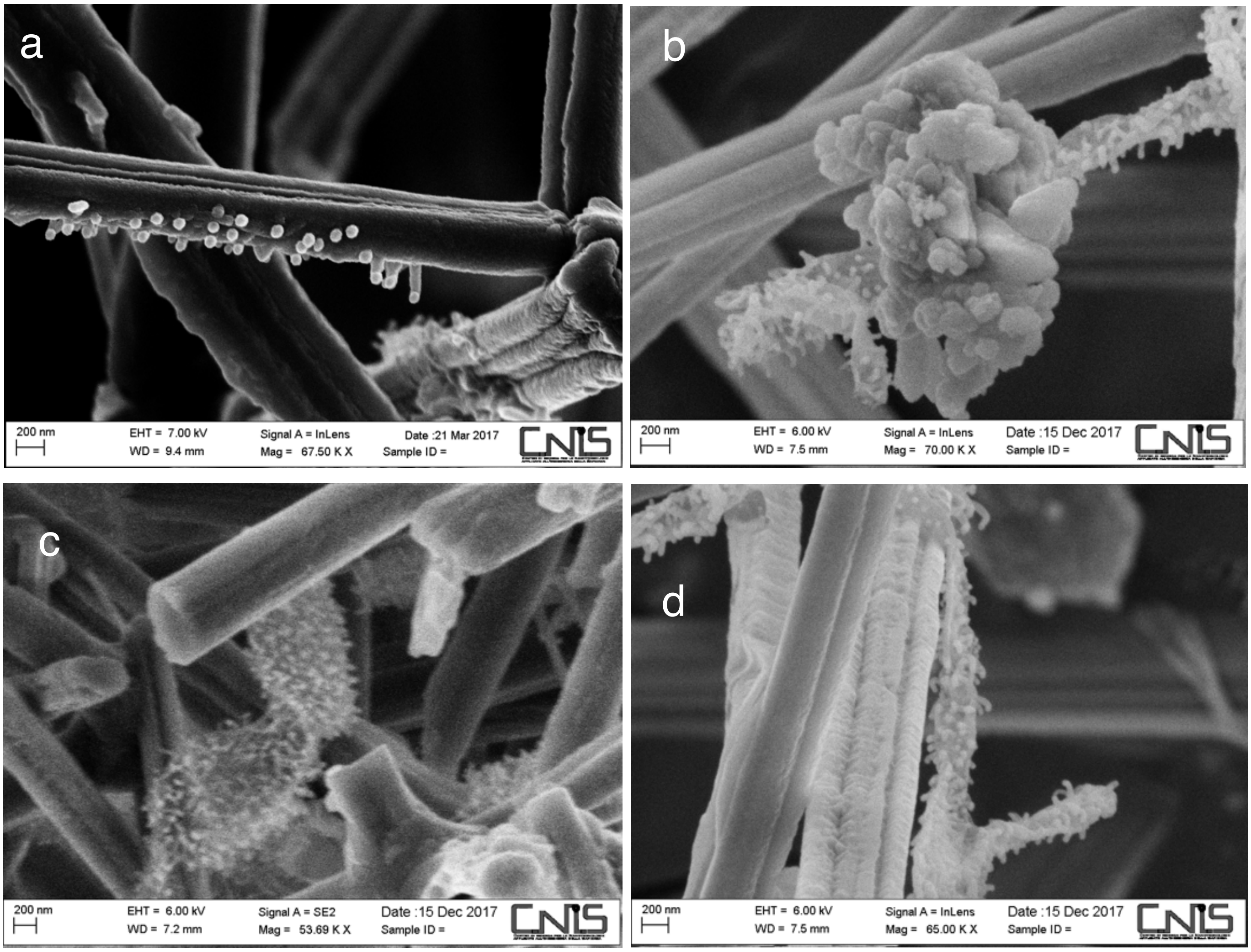

Figure S3.
SEM analysis of moonmilk isolated from the *Stanza degli Scudi*. (a) Magnification of the same microorganism of Fig. 3d and 3e wrapping a $CaCO_3$ nanofiber; the sample was taken in March 2017. (b, c and d) Magnification of the microorganism in Fig. 3 h sampled in December 2017.

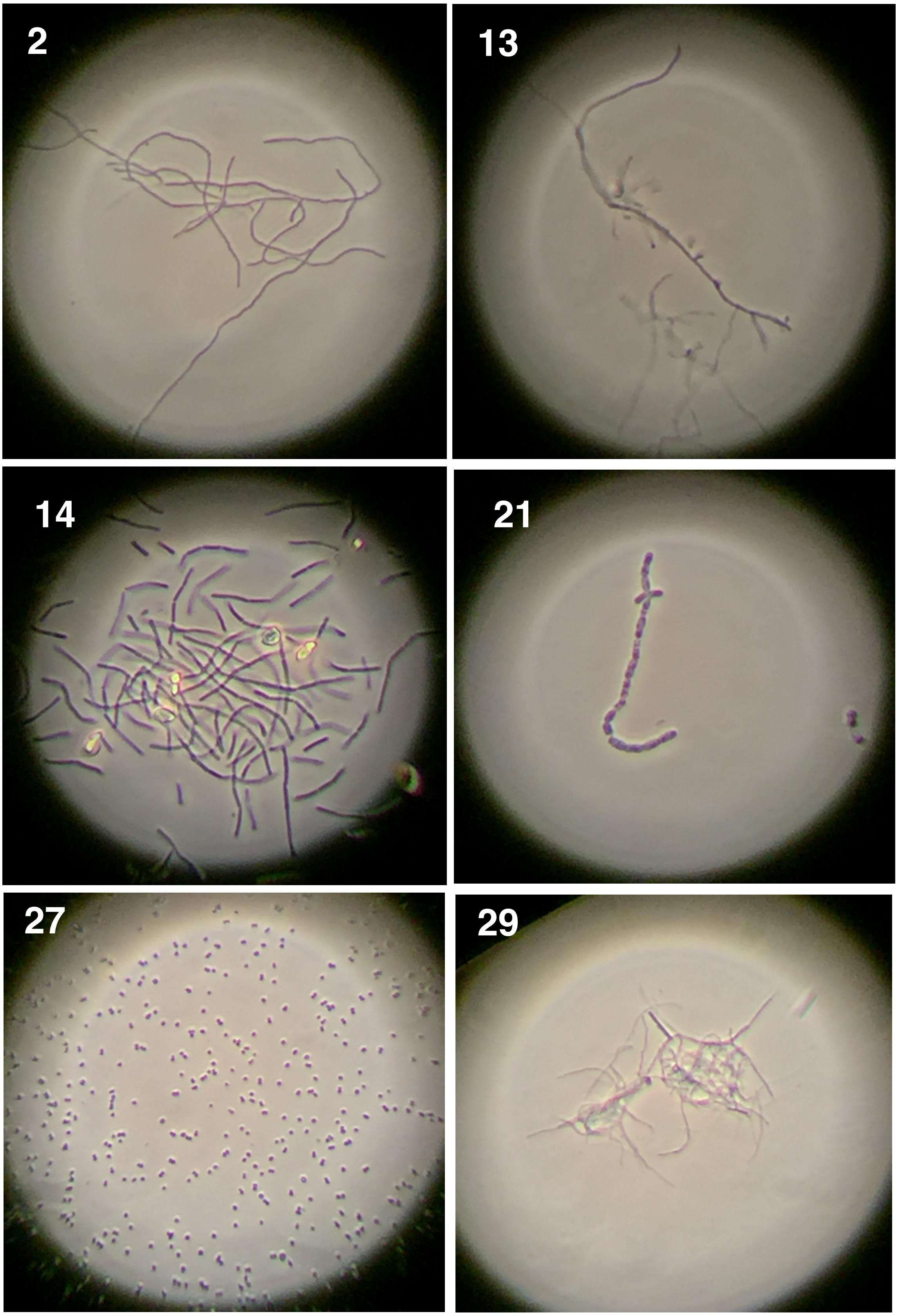

Figure S4.
Examples of bacterial strains isolated from moonmilk in the *Stanza degli Scudi*. The strains were grown in complete medium and cultured at 18 °C. *Streptomyces* sp. TR2, *Streptomyces* 13, *Bacillus* 14, *Bacillus mycoides* 21, cocci 27 and *Streptomyces* 29. Pictures were taken using an Axio Star Zeiss microscope, magnification 40×.

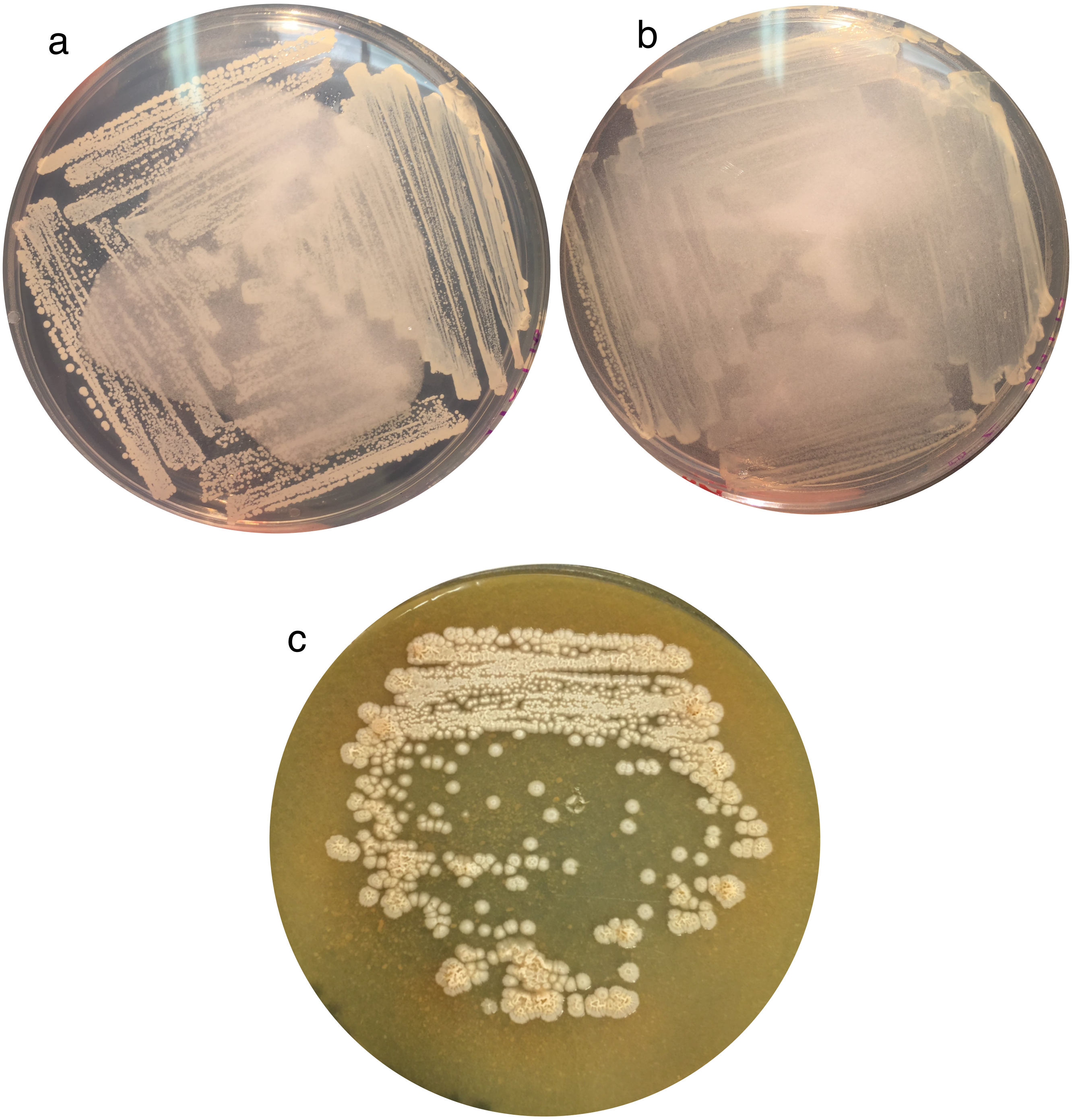

Figure S5.
Dissolution and deposition of calcium carbonate activity on plates of two strains isolated from moonmilk. a) Strain 27 was streaked on B4-C plates and during growth, the cells dissolved $CaCO_3$, rendering the medium transparent. b) As a control, strain 14, a *Bacillus* sp., was not able to dissolve $CaCO_3$, and the plate remained opaque. c) *Streptomyces* sp. TR2 was streaked on YPD supplemented with urea, and cells were observed directly with SEM (see Fig. 4f).

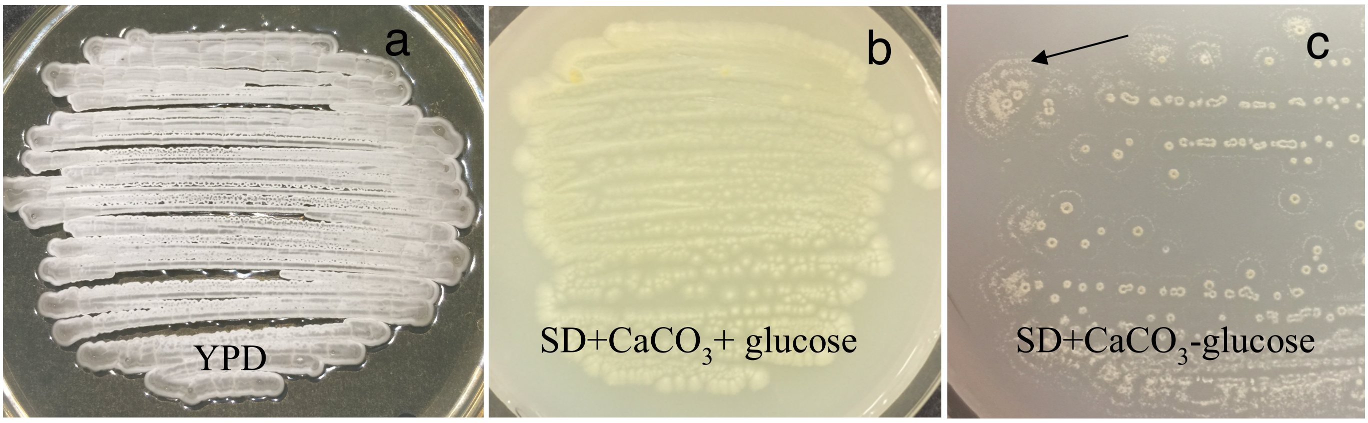

Figure S6.
Growth of *Streptomyces* colony 2 isolated from the *Stanza degli Scudi* in March 2017. (a) Complete medium YPD. (b) Synthetic medium supplemented with $CaCO_3$ and glucose. (c) Synthetic medium supplemented with $CaCO_3$ but without glucose. The black flag indicates where the SEM sample of Fig. 4 (d), (e), (f) was taken.

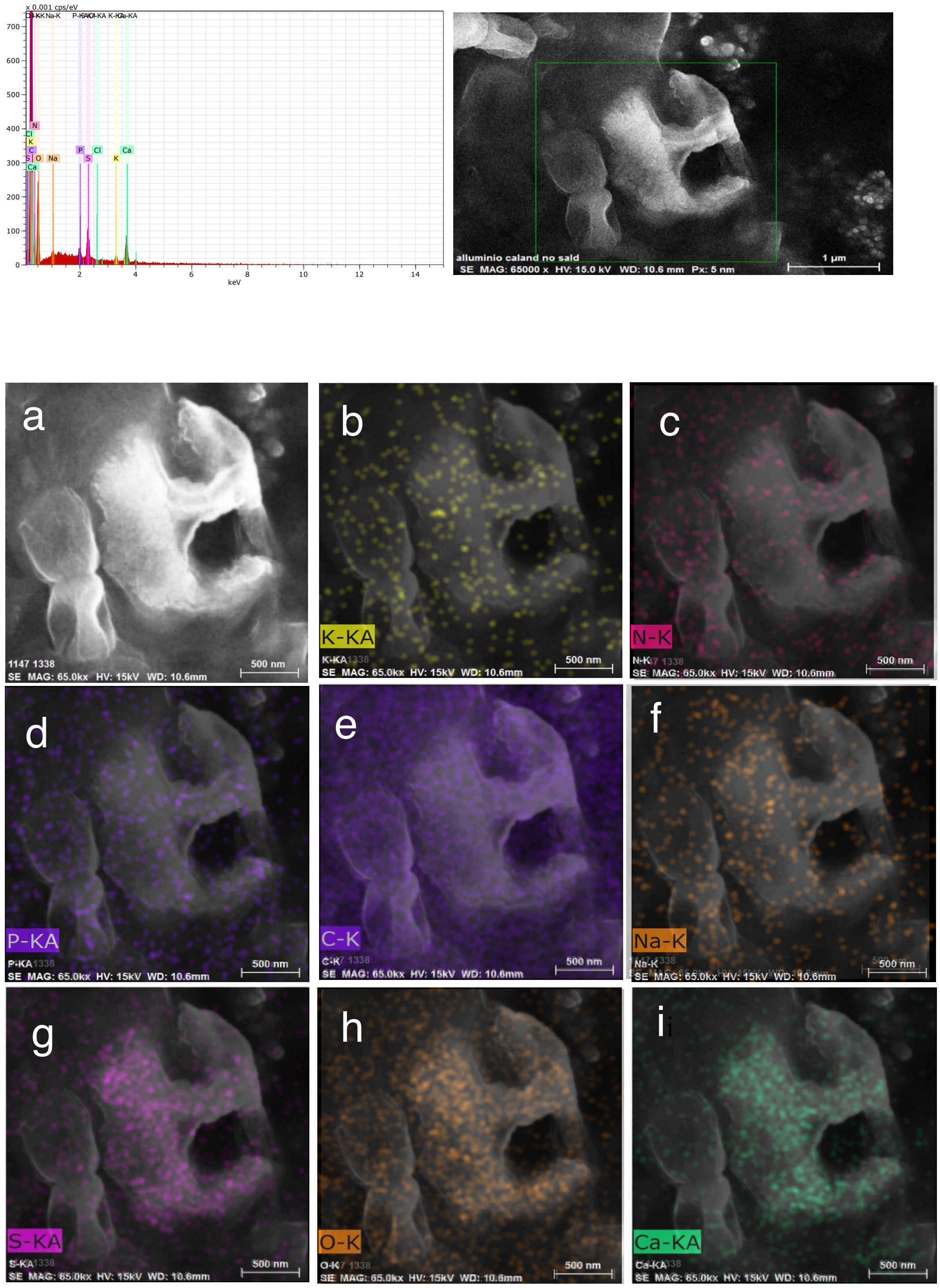

Figure S7.

SEM analysis of a pure culture of *Streptomyces* 2 grown in SD medium without glucose. The atomic composition of the deposit in Figure 4e suggests a calcium sulfate composition (S5g-i).

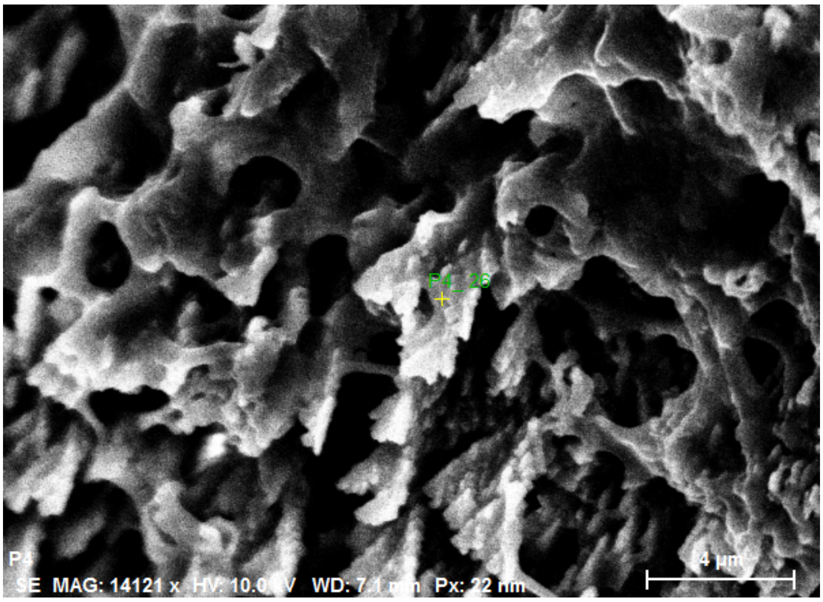

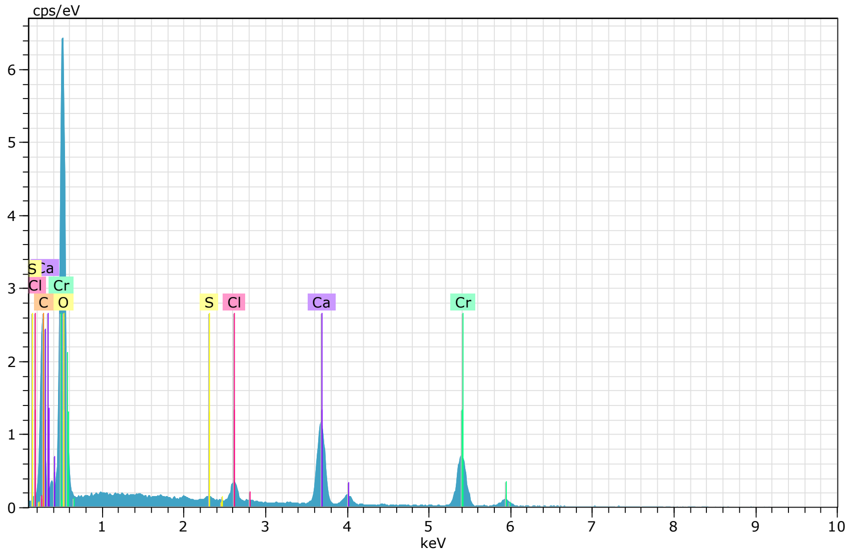

Spectrum: P4_ 26

El	AN	Series	unn. C [wt.%]	norm. C [wt.%]	Atom. C [at.%]	Error (1 Sigma) [wt.%]
O	8	K-series	39.05	58.00	63.57	5.03
C	6	K-series	11.78	17.50	25.55	1.77
Ca	20	K-series	14.85	22.06	9.65	0.56
Cl	17	K-series	1.41	2.09	1.03	0.09
S	16	K-series	0.24	0.35	0.19	0.04
Cr	24	K-series	0.00	0.00	0.00	0.00
		Total:	67.33	100.00	100.00	

Figure S8
SEM analysis of a pure culture of *Streptomyces* 2 grown in YPD medium with urea. The atomic composition of the deposit in Figure 4f suggests a calcium carbonate composition.

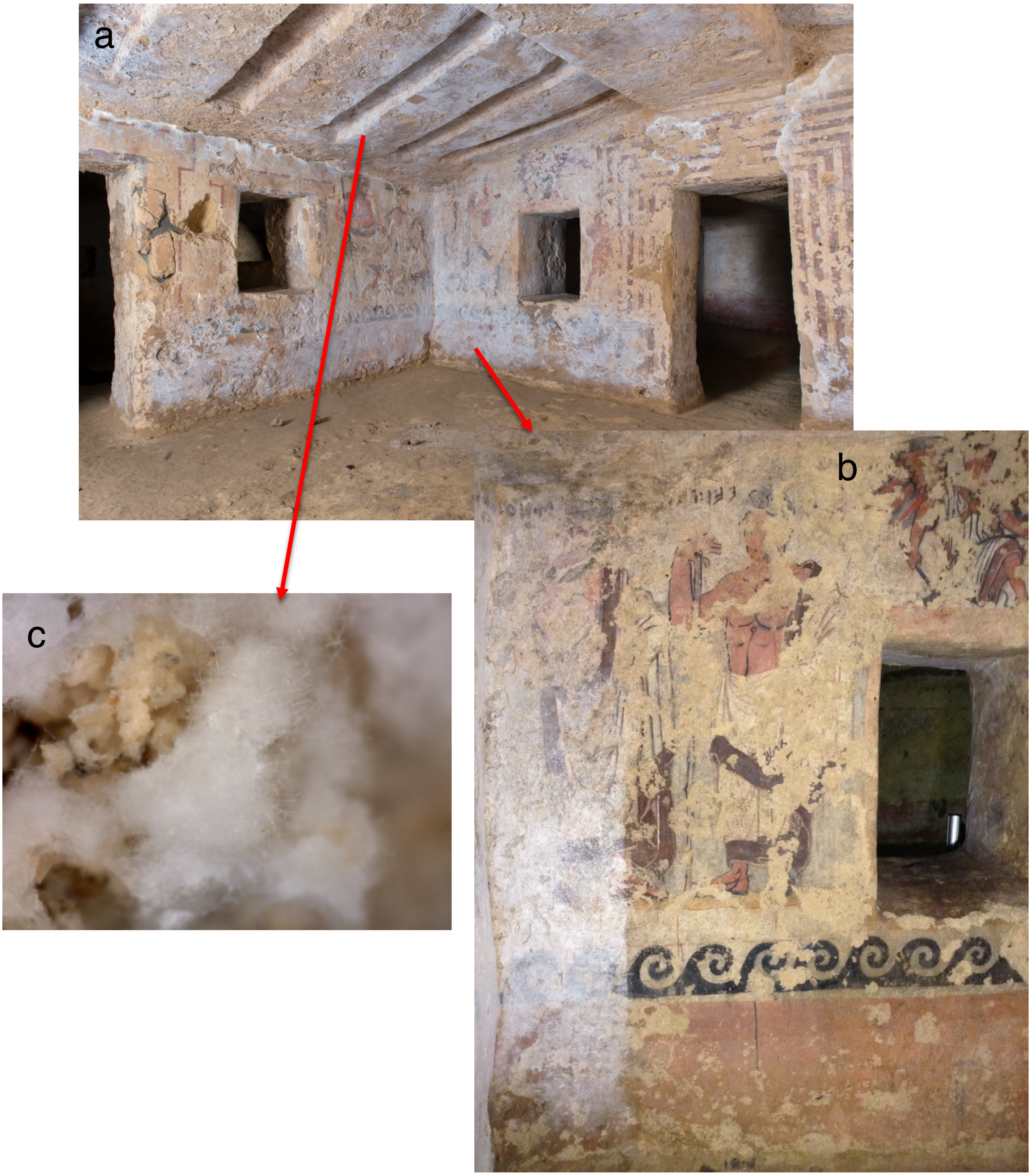

Figure S9.
Pictures of the white deposit in the central atrium in the *Tomba degli Scudi* (a) General view of the central atrium before restoration. (b) The picture shows the left part (red arrow) of the western wall during restoration; in this part of the wall the white deposit covering the frescoes was soft. (c) The soft white deposit on the roof (red arrow) was not removed during restoration; it is still in place and corresponds to moonmilk. The pictures were taken directly with a video microscope. Photographer: Domenico Ventura.

Table S1. The closest relatives bacterial species of the colony 2 isolated in *Stanza degli Scudi*.

Strain	Query cover	Identity	Max score	Total score	Accession number
Streptomyces sp. MM122 16S ribosomal RNA gene, partial sequence	100%	99%	1247	1247	KU714879.1
Streptomyces sp. strain KMSt5 16S ribosomal RNA gene, partial sequence	100%	99%	1247	1247	KX950877.1
Streptomyces sp. E1N444 16S ribosomal RNA gene, partial sequence	100%	99%	1247	1247	KX279644.1
Streptomyces sp. E5N142 16S ribosomal RNA gene, partial sequence	100%	99%	1247	1247	KX279565.1
Streptomyces badius strain DB-1 16S ribosomal RNA gene, partial sequence	100%	99%	1247	1247	KU886090.1
Streptomyces sp. MOLA 1484 16S ribosomal RNA gene, partial sequence	100%	99%	1247	1247	KM273899.1
Streptomyces flavogriseus strain Nr_2 16S ribosomal RNA gene, partial sequence	100%	99%	1247	1247	KT714128.1
Streptomyces flavogriseus strain ACTK2 16S ribosomal RNA gene, partial sequence	100%	99%	1247	1247	KC990785.1

Table S2. Features defining *genus/species* in Western and Northern walls samples.

**Taxon**	**W %**	**N %**
k__Bacteria;p__Actinobacteria;c__Actinobacteria;o__Actinomycetales;f__Nocardioidaceae;g__Actinopoly-morpha;s__	0,3678823	0
k__Bacteria;p__Actinobacteria;c__Actinobacteria;o__Actinomycetales;f__Nocardioidaceae;g__Krib-bella;s__	0	0,2336085
k__Bacteria;p__Actinobacteria;c__Actinobacteria;o__Actinomycetales;f__Pseudonocardiaceae;g__Pseu-donocardia;s__	5,6941779	3,4729793
k__Bacteria;p__Chlamydiae;c__Chlamydiia;o__Chlamydiales;f__Parachlamydiaceae;g__Candidatus Pro-tochlamydia;s__	0	0,1401651
k__Bacteria;p__Firmicutes;c__Bacilli;o__Bacillales;f__Bacillaceae;g__Bacillus;s__muralis	0	0,7475471
k__Bacteria;p__Firmicutes;c__Bacilli;o__Bacillales;f__Paenibacillaceae;g__Paenibacillus;__	0	0,7008254
k__Bacteria;p__Nitrospirae;c__Nitrospira;o__Nitrospirales;f__Nitrospiraceae;g__Nitrospira;s__	0,6078055	2,1491979
k__Bacteria;p__Planctomycetes;c__Planctomycetia;o__Gemmatales;f__Gemmataceae;g__Gemmata;s__	0,3039028	0,7942688
k__Bacteria;p__Planctomycetes;c__Planctomycetia;o__Pirellulales;f__Pirellulaceae;g__Pirellula;s__	0	0,4983647
k__Bacteria;p__Proteobacteria;c__Alphaproteobacteria;o__Rhizobiales;f__Bradyrhizobiaceae;g__Bradyrhi-zobium;s__	0,4158669	0,3737736
k__Bacteria;p__Proteobacteria;c__Alphaproteobacteria;o__Rhizobiales;f__Hyphomicrobiaceae;g__Hy-phomicrobium;s__	2,9590531	2,4295281
k__Bacteria;p__Proteobacteria;c__Alphaproteobacteria;o__Rhizobiales;f__Hyphomicrobiaceae;g__Pe-domicrobium;s__	0,5758157	0
k__Bacteria;p__Proteobacteria;c__Alphaproteobacteria;o__Rhizobiales;f__Hyphomicrobi-aceae;g__Rhodoplanes;s__	0,5118362	1,8532939
k__Bacteria;p__Proteobacteria;c__Alphaproteobacteria;o__Sphingomonadales;f__Sphingomona-daceae;g__Sphingopyxis;s__alaskensis	0,1919386	0
k__Bacteria;p__Proteobacteria;c__Betaproteobacteria;o__Burkholderiales;f__Comamonadaceae;g__Me-thylibium;__	0,9916827	1,1991902
k__Bacteria;p__Proteobacteria;c__Deltaproteobacteria;o__Bdellovibrionales;f__Bdellovibri-onaceae;g__Bdellovibrio;s__	0	0,4672169
k__Bacteria;p__Proteobacteria;c__Gammaproteobacteria;o__Legionellales;f__Coxiellaceae;g__Aqui-cella;s__	0,1599488	0
k__Bacteria;p__Proteobacteria;c__Gammaproteobacteria;o__Xanthomonadales;f__Sinobacter-aceae;g__Nevskia;s__	0,0799744	0
k__Bacteria;p__Proteobacteria;c__Gammaproteobacteria;o__Xanthomonadales;f__Sinobacter-aceae;g__Steroidobacter;s__	1,0236724	0,7163993
k__Bacteria;p__Verrucomicrobia;c__[Spartobacteria];o__[Chthoniobacterales];f__[Chthoniobacter-aceae];g__Candidatus Xiphinematobacter;s__	0	0,6852515
% on the total	13,883557	16,46161
